# Integration of Transcriptomic and Metabolomic Profiles Provides Insights into the Influence of Nitrogen on Secondary Metabolism in *Fusarium sacchari*

**DOI:** 10.3390/ijms241310832

**Published:** 2023-06-29

**Authors:** Yixue Bao, Zhenyue Lin, Wei Yao, Sehrish Akbar, Wenfeng Lin, Charles A. Powell, Jianlong Xu, Muqing Zhang

**Affiliations:** 1 State Key Lab for Conservation and Utilization of Subtropical Agric-Biological Resources & Guangxi Key Lab for Sugarcane Biology & Academy of Sugarcane and Sugar Industry, Guangxi University, Nanning 530004, China; baoyixue57319@163.com (Y.B.); linzhenyue@mju.edu.cn (Z.L.); yaoweimail@163.com (W.Y.); sehrishakbar746@gmail.com (S.A.); lin1224540633@163.com (W.L.); 2 IFAS Indian River Research and Education Center, University of Florida, Fort Pierce, FL 34945, USA; capowell@ufl.edu; 3 Hainan Yazhou Bay Seed Laboratory, National Nanfan Research Institute (Sanya), Chinese Academy of Agricultural Sciences, Sanya 572025, China; 4 Institute of Crop Sciences, Chinese Academy of Agricultural Sciences, Beijing 100081, China

**Keywords:** nitrogen, secondary metabolism, *Fusarium sacchari*, gibberellin, mycotoxin

## Abstract

Nitrogen availability might play an essential role in plant diseases by enhancing fungal cell growth and influencing the expression of genes required for successful pathogenesis. Nitrogen availability could modulate secondary metabolic pathways as evidenced by the significant differential expression of several core genes involved in mycotoxin biosynthesis and genes encoding polyketide synthase/nonribosomal peptide synthetases, cytochrome P450 and carbohydrate-active enzymes in *Fusarium sacchari*, grown on different nitrogen sources. A combined analysis was carried out on the transcript and metabolite profiles of regulatory metabolic processes and the virulence of *Fusarium sacchari* grown on various nitrogen sources. The nitrogen regulation of the gibberellin gene cluster included the metabolic flux and multiple steps of gibberellin synthesis. UHPLC-MS/MS-based metabolome analysis revealed the coordination of these related transcripts and the accumulation of gibberellin metabolites. This integrated analysis allowed us to uncover additional information for a more comprehensive understanding of biological events relevant to fungal secondary metabolic regulation in response to nitrogen availability.

## 1. Introduction

The critical factors affecting fungal growth are nitrogen sources that affect the growth and synthesis of secondary metabolites, including mycotoxins. Nitrogen commonly limits plant growth in terrestrial ecosystems and affects competitive interactions within plant-microbe communities, including pathogen and symbiotic microorganisms [1]. Although nitrogen fertilizers improve the nutrient status of plant hosts, they can also promote disease by affecting physiological and biochemical balances in plants [2,3]. However, the overuse of nitrogen fertilizers and the inadequate timing of fertilizer application can promote plant disease outbreaks [4].

Pokkah boeng disease (PBD) is responsible for devastating sugarcane yield loss worldwide, especially in China, India, Malaysia, and South Africa [5,6]. The *Fusarium fujikuroi* species complex (FFSC) is responsible for the PBD of sugarcane [7]. *Fusarium sacchari* (*F. sacchari*) was the dominant species, accounting for over 50% of the PBD-isolated strains [7]. This species produces several harmful mycotoxins, including fusarubin, bikaverin, fusaric acid, and trichothecene deoxynivalenol, which contaminate grains and render them unsafe for human or livestock consumption [8]. The production of secondary metabolites (SMs), such as pathogenicity factors released by the FFSC, might be essential for these fungi’s day-to-day survival and metabolism. SMs are not essential for life but can provide an advantage under natural conditions and contribute to the virulence of the pathogen [9]. Gene clusters for secondary metabolite biosynthesis have been documented in bioinformatics studies, but a detailed characterization of secondary metabolite biosynthetic pathways and global regulation is unavailable [10].

This study was conducted to provide insight into the secondary metabolites’ production of *F. sacchari* and morphological alterations that occur in response to nitrogen availability [11,12,13]. A comprehensive analysis of secondary metabolism in *F. sacchari* was performed using RNA-Seq and UHPLC-MS/MS analyses. The expression profiles of genes were evaluated to be involved in secondary metabolite biosynthesis and quantify the production of secondary metabolites in response to nitrogen availability. The data generated will contribute to filling up the gaps in the previous studies to some extent.

## 2. Results

### 2.1. Transcriptome Analysis

To identify the critical genes in response to nitrogen availability and the production of secondary metabolites in *F. sacchari*, we performed the transcriptomic analyses of FsCNO-1, including ‘urea’, ‘NaNO_3_’, and ‘(NH_4_)_2_SO_4_’ group. We obtained 67,379,846 clean reads, with an average GC content of 52.73%. The raw reads were submitted to the GenBank Sequence Read Archive (SRR18439752, SRR18439753, and SRR18439754). Approximately 94.93% of the clean reads mapped to the *F. sacchari* CNO-1 genome and more than 93.65% could be uniquely mapped.

Compared to the NaNO_3_, 1036 DEGs were identified in urea, including 435 up-regulated and 601 down-regulated, and 1390 DEGs in (NH_4_)_2_SO_4_, including 702 up-regulated and 688 down-regulated. However, compared to the (NH_4_)_2_SO_4_, 1516 DEGs were detected in urea, including 653 up-regulated and 863 down-regulated. A total of 132 genes were differentially expressed in the three comparison groups. These DEGs might play a role in response to nitrogen availability (Figure S1). The KEGG pathways were highly enriched in ‘urea vs. NaNO_3_’, including ‘Nitrogen metabolism’, ‘Valine, leucine and isoleucine degradation’, and ‘Steroid biosynthesis’. However, ‘Nitrogen metabolism’, ‘beta-Alanine metabolism’, and ‘Tyrosine metabolism’ KEGG pathway were highly enriched in ‘(NH_4_)_2_SO_4_ vs. NaNO_3_’. ‘Pentose and glucuronate interconversions’, ‘Ascorbate and aldarate metabolism’, and ‘Valine, leucine, and isoleucine degradation’ KEGG pathway were highly enriched in ‘urea vs. (NH_4_)_2_SO_4_’ (Table S1 and Figure S2).

### 2.2. Gibberellins Production

**Figure 1:**
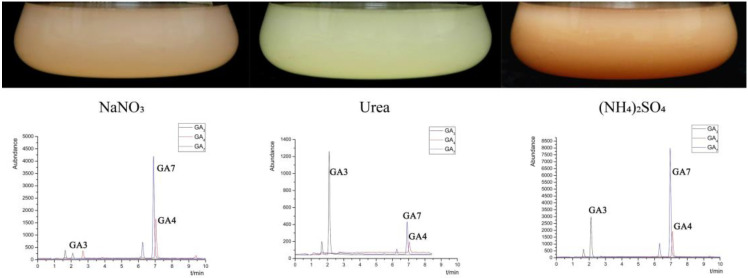
Pigmentation characteristics and the production of gibberellin in FsCNO-1 cultured in different nitrogen availability.

FsCNO-1 strains displayed variation in pigmentation three days after inoculation with different nitrogen treatments (Figure 1). We investigated the ability of the FsCNO-1 to produce gibberellins (GA7, GA3, and GA4 were the main gibberellins). GA7 and GA4 accumulated up to 10,000 and 6000 µg/L after culturing for three days in (NH_4_)_2_SO_4_ and NaNO_3_ culture conditions. Meanwhile, cultures grown with urea had GA7 and GA4 levels at 600 µg/L. GA3 produced about 3-fold more than GA4 and GA7 when cultured in the urea, whereas GA7 was approximately 2.0-fold more than what GA3 produced when cultured in (NH_4_)_2_SO_4_ (Figure 1).

### 2.3. Genes Associated with GA Production in FsCNO-1

**Figure 2:**
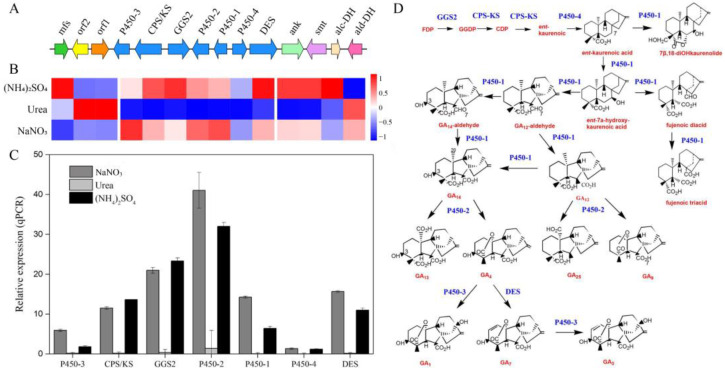
Gene clusters and their expressions of gibberellin in FsCNO-1 cultured in different nitrogen availability. (**A**) Gibberellin and flanking gene cluster in FsCNO-1. Arrows show the orientation of transcription. Gibberellin genes are shown in blue and other colors are shown flanking the GA cluster. (**B**) Heatmap analysis of expression data derived from RNA-Seq for the gibberellin gene cluster after culturing in different nitrogen treatments. (**C**) Relative expression of gibberellin core genes validated by qRT-PCR. (**D**) Gibberellin biosynthetic pathway in the *F. sacchari*.

The GA gene cluster contains a total of seven functional genes, including *DES* (*FSAC_09644*), *P450-4* (*FSAC_12979*), *P450-1* (*FSAC_10060*), *P450-2* (*FSAC_08504*), *GGS2* (*FSAC_05737*), *CPS/KS* (*FSAC_08126*), and *P450-3* (*FSAC_03987*) which were present in FsCNO-1. In addition, genes encoding the ankyrin (*ank*), sugar membrane transporter (*smt*), alcohol (*alc-DH*), and aldehyde dehydrogenase (*ald-DH*) were located upstream of the GA cluster (Figure 2A). Transcriptome data revealed that the expression of genes within and flanking the GA cluster had high transcriptional plasticity in response to nitrogen availability (Figure 2B). We performed qRT-PCR to assess the expression of GA cluster genes and found that the calculated fold changes were consistent with those determined by RNA-seq (Figure 2C).

Noticeably, growth in media having urea as the nitrogen source completely repressed the expression of the GA cluster core genes, which had an overall effect of decreasing the pool of GA cluster transcripts, limiting GA metabolic flux. In particular, genes encoding cytochrome P450 monooxygenase (*P450-3*), geranylgeranyl diphosphate synthase (*GGS2*), and/or ent-copalyl diphosphate/ent-kaurene synthase (*CPS/KS*), cytochrome P450 monooxygenase *(P450-2)*, GA14-synthase (*P450-1*), and GA4 desaturase (*DES*) showed significantly higher transcript levels on either (NH_4_)_2_SO_4_ or NaNO_3_, as compared to urea. In addition, several intermediates and final biosynthetic steps are catalyzed by *P450-2* and *DES* (Figure 2D) [14]. Although the *P450-3* gene expression was accompanied by relatively high levels of GA3 production in (NH_4_)_2_SO_4_ media, only low production levels were seen for growth on NaNO_3_. Our results show that the total GA production largely corresponded with the gene expression levels. Six out of seven GA genes in FsCNO-1 were regulated by nitrogen, except for ent-kaurene oxidase (*P450-4*), which showed only faint expression and remained low (fold change < 1.0) regardless of the nitrogen source. However, the expression levels of seven GA-flanking genes were highly variable (Figure 2B). Genes flanking the GA cluster are often regulated by a transcriptional regulator in the cluster itself that could co-regulate the transcription of cluster genes [15]. However, our data could not define how the expression of GA-flanking genes is affected by the nitrogen source or whether these genes coordinate the expression of GA cluster genes during GA synthesis in response to specific nitrogen signals.

### 2.4. Genes Related to Secondary Metabolite Production in FsCNO-1

**Figure 3:**
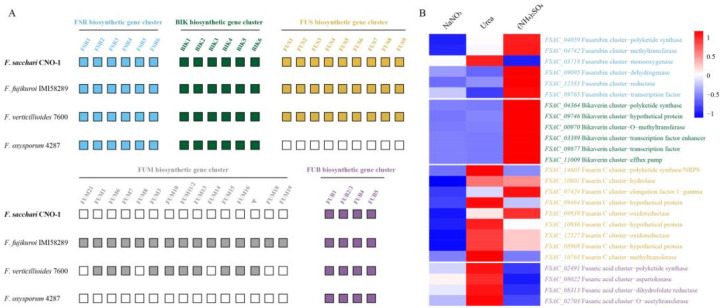
Nitrogen influences the expression of mycotoxin biosynthesis genes in FsCNO-1. (**A**) Comparison of FSR, BIK, FUS, FUM, and FUB biosynthetic gene clusters between four Fusarium species. (**B**) Heatmap clustering of the gene expression profiles of core enzyme genes associated with mycotoxin biosynthesis. Note: FSR—fusarubin, BIK—bikaverin, FUS—fusarin C, FUM—fumonisin, FUB—fusaric acid, and Ψ indicates the pseudogene. The white box represent no detected cluster genes.

**Mycotoxin synthesis.** To enhance our understanding of secondary metabolism events associated with the nitrogen regulation of biosynthetic gene clusters, we analyzed FSR, BIK, FUB, FUS, and FUM biosynthetic gene clusters related to mycotoxin synthesis. FSR mediates the biosynthesis of fusarubin, BIK for bikaverin, FUB for fusaric acid, FUS for fusarin C, and FUM for fumonisin. We identified complete FSR, BIK, FUS, and FUB biosynthetic gene clusters in FsCNO-1 genomes, lacking a complete FUM gene cluster (Figure 3A).

Based on the clustering analysis, bikaverin, and fusarubin-associated genes were expressed to significantly higher levels in (NH_4_)_2_SO_4_ media relative to urea or NaNO_3_ media. Specifically, the genes were related to bikaverin synthesis, including *FSAC_04364* (polyketide synthase), *FSAC_09746* (hypothetical protein), *FSAC_00970* (O-methyltransferase), *FSAC_03389* (transcription factor enhancer), *FSAC_09877* (transcription factor), and *FSAC_11009* (efflux pump), and showed a markedly induced expression in (NH_4_)_2_SO_4_ media. Moreover, the pattern of the fusarubin synthesis is similar. The fusarubin biosynthetic gene cluster mainly contains six functional genes (*FSAC_04059*, *FSAC_04742*, *FSAC_03718*, *FSAC_09095*, *FSAC_12585*, and *FSAC_09765*), and most genes also showed an up-regulated expression in (NH_4_)_2_SO_4_ media (Figure 3B). Significantly, in contrast to the utterly repressed expression pattern of GA genes with urea as the nitrogen source, the expression of genes associated with fusarin C (*FSAC_14605*, *FSAC_10601*, *FSAC_07424*, *FSAC_09464*, *FSAC_09939*, *FSAC_10936*, *FSAC_12527*, *FSAC_08908*, and *FSAC_10763*) and fusaric acid (*FSAC_02491*, *FSAC_08022*, *FSAC_08313*, and *FSAC_02703*) production was distinctly activated on urea (Figure 3B). Furthermore, fusaric acid-related gene expression was obviously higher than other mycotoxins (Table 1).

**Figure 4:**
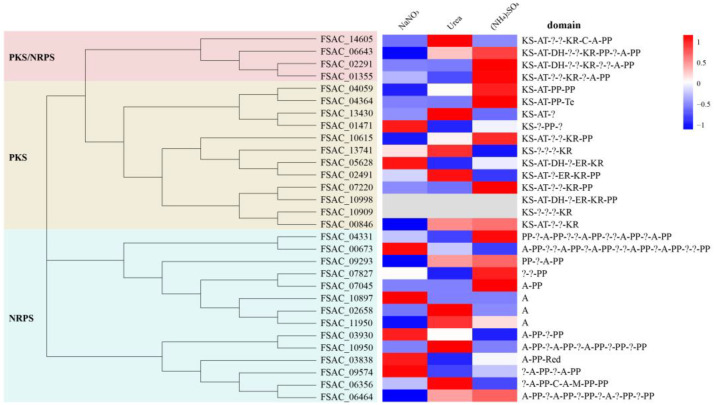
Nitrogen regulation of PKS/NRPS expression in FsCNO-1. The phylogenetic tree (**left panel**) of PKSs and NRPSs encoded by FsCNO-1 sequences; Expression patterns of PKS- and NRPS-associated genes in different nitrogen (**right panel**). Note: KS, keto synthase; AT, acyltransferase; DH, dehydratase; ER, enoyl reductase; KR, keto reductase; PP, acyl carrier protein; A, adenylation domain; C, condensation domain; Te, thioesterase domain; ?, unknown protein.

**PKS/NRPS.** Sixteen putative PKS genes, fourteen NRPS genes, and four PKS-NRPS hybrid genes were identified in FsCNO-1 (Table S2). A prediction tool identified a complete set of conserved domains comprising each PKS or NRPS gene (Figure 4). Based on Clustal W v2.0 and MEGA v7.0 software, a phylogenetic tree of the PKSs and NRPSs protein sequences was performed using the neighbor-joining (NJ) method. In addition, in the expression of PKSs and NRPS genes, significant variation was detected for the FsCNO-1 transcriptome. Only two putative PKS genes (*FSAC_10998* and *FSAC_10909*) were silent and could not express, and the other twenty-eight genes showed distinctly different expression patterns for growth on different nitrogen sources (Figure 4).

**Cytochrome P450.** Cytochrome P450 is a member of the CYP superfamily of heme-containing monooxygenases involved in the production and/or modification of diverse compounds [16]. We performed a batch BLAST search against the InterPro database and identified 156 putative cytochrome P450-coding genes in FsCNO-1 (Table S3). Among these genes, 111 genes had (log2FC > 1.0, *p* < 0.05) different expressions according to our RNA-Seq analysis, which suggested that nitrogen availability could influence the expression of most cytochrome P450 genes. Among these DEGs, the transcription levels of most DEGs were significantly down-regulated in NaNO_3_ media, whereas many DEGs were up-regulated in (NH_4_)_2_SO_4_ and urea media (Figure S3).

Cytochrome P450 has a multifunctional role in fungi-like secondary, primary, xenobiotic, and drug metabolism. RNA-seq analysis was performed to examine changes in the expression of several representative P450 genes with known functions. As expected, a series of P450-encoding genes were assigned to the secondary metabolite class, involved in the biosynthesis of trichothecene and sterigmatocystin. Interestingly, cytochrome P450-Pdm (*FSAC_01874*, *FSAC_10736*, *FSAC_07317*, and *FSAC_13209*) plays a defensive role against factors secreted by the host and increases fungal pathogenicity, indicating a significant expression induction when (NH_4_)_2_SO_4_ or urea was used as the nitrogen source (Table 2).

**CAZyme families.** CAZyme families in fungi orchestrate the bioactivity and metabolism of small molecules that comprise various glycosylated compounds (e.g., glycolipids, glycoproteins, lignin precursors, and secondary metabolites), including glycoside hydrolases (GHs), glycosyl transferases (GTs), polysaccharide lyases (PLs), carbohydrate esterases (CEs), auxiliary activities (AAs), and carbohydrate-binding modules (CBMs) [17]. A total of 786 CAZY genes were detected in FsCNO-1, including 315 GHs (315), 174 CEs (174), 120 AAs, 109 GTs, 48 CBMs, and 20 PLs.

**Figure 5:**
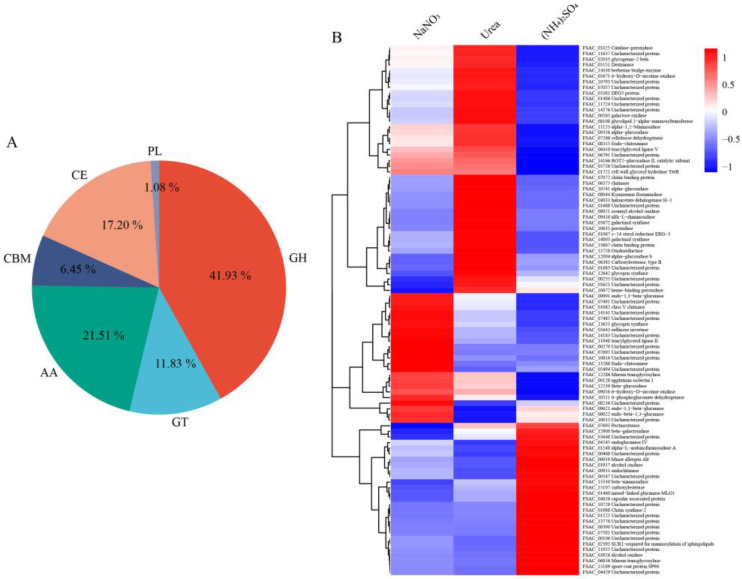
Nitrogen regulation of the expression of CAZy family genes in FsCNO-1. (**A**) Differentially expressed genes related to CAZy families were represented by their classes. (**B**) Heatmap clustering analysis of differentially expressed genes related to CAZy enzyme.

Moreover, 93 CAZy genes were differentially expressed among the nitrogen treatments, including 39 GHs, 11 GTs, 20 AAs, 6 CBMs, 16 CEs, and 1 PLs (Figure 5A). Next, we performed hierarchical clustering and heat map analysis, which found significant differences in FsCNO-1 grown on urea (Figure 5B). A large proportion of alpha-glucosidase, chitinase, and galactinol synthase genes could be induced with a high expression of growth on urea when compared with NaNO_3_ or (NH_4_)_2_SO_4_. Overall, the type of nitrogen source significantly affected the expression of the prominent CAZy families in our studies and was mainly involved in regulating the biological processes related to SMs in *Fusarium*, such as mycotoxin, PKS/NRPS, and cytochrome P450.

## 3. Discussion

**Figure 6:**
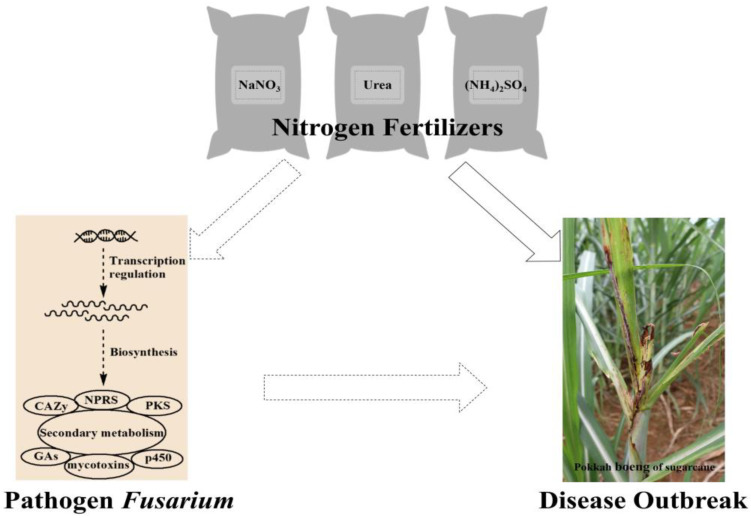
The diagram of relationships between the nitrogen fertilizers, pathogen Fusarium, and pokkah boeng disease outbreak.

Pokkah boeng disease has spread rapidly in China during all seasons in recent years. Guangxi, the most extensive sugarcane planting and production area in China, was first reported to be seriously affected due to PBD [18]. Here, we performed the RNA-seq and UHPLC-MS/MS analysis of FsCNO-1, which are reported to be the significant representative pathogens of PBD [7]. Nitrogen was associated with *F. sacchari* cell growth speed and plant disease severity. The expression of genes required for successful pathogenesis was affected, such as those involved in nitrogen assimilation, nitrogen sensing and regulation, and potential virulence- and pathogenicity-associated functions. Many genes associated with secondary metabolism were also affected by the nitrogen source [13]. Several regulators were found to be associated with specific biological processes related to nitrogen utilization [19,20,21]. The genes associated with secondary metabolite synthesis in FsCNO-1 were investigated in silico. Nitrogen availability could modulate secondary metabolic pathways as evidenced by the significant differential expression of cluster genes involved in mycotoxin biosynthesis and genes encoding polyketide synthase/nonribosomal peptide synthetases, cytochrome P450, and carbohydrate-active enzymes in *F. sacchari* grown on different nitrogen sources. The nitrogen regulation of the gibberellin gene cluster included metabolic flux and multiple steps of gibberellin synthesis. RNA-seq and UHPLC-MS/MS analysis revealed the coordination of these related transcripts and the accumulation of gibberellin metabolites. For sugarcane, the pathogenic FsCNO-1 of PBD resulted in the significant inhibition of leaf NR, GS, and GOGAT activities, decreased NO^3−^-N and NH^4+^-N assimilation rates, and continued to promote RNA degradation, thereby reducing ribosome and protein synthesis. Glutamine plays an important protective role by regulating the biosynthesis of proline and polyamines. The metabolic pathways of cyanamide, glutamic acid, proline, tyrosine, and arachidonic acid actively respond to the stress of pathogens [22]. The results provide a scientific basis for the formulation of technical regulations for the rational fertilization of sugarcane (Figure 6).

FsCNO-1 cultured in different nitrogen sources showed differences in the production of pigments associated with secondary metabolites. However, the detailed mechanisms still need to be clarified. Notably, many genes central to producing fusarubin, bikaverin, fusaric acid, and fusarin C had an altered expression according to the different nitrogen sources, which strongly implied the existence of specific fungal metabolites that might play a role in mycotoxin production. Previous studies revealed a correlation between nitrogen availability and production of other SMs (e.g., gibberellins, mycotoxin, and CAZYmes, etc.) by *Fusarium* (Figure 6), such as that arising from carotenoids, the PKS-derived mycelial reddish pigments bikaverin, and fusarubins [9], and the brown or black melanin pigments [23]. In this study, the results showed that the appearance of the reddish pigment observed in cultured ammonium as the sole nitrogen source, was coincident with the ammonium-induced abundant expression of BIK in FsCNO-1.

The expression profile of CAZyme family genes suggested that the expression of AA and CE genes induced by growth on ammonium could preferentially contribute to fungal pathogenicity and fungal biomass production, as consistent with our previous study [13]. A diverse arsenal of CAZymes might thus be needed to access the plant xylem and to breach plant defense structures (e.g., xyloses and pectin gels) [17,24]. Genes encoding cytochrome P450 appear in gene clusters with neighboring PKS or mycotoxin genes and encode proteins that can modify or customize the polyketide backbone to form functional secondary metabolites [25].

*Fusarium* can generate a vast array of bioactive secondary metabolites, which are linked to the biosynthetic genes responsible for their production [26], such as PKS and NRPS, although many of these genes have unknown products [27,28]. The PKS and NRPS genes provide a basis for further studies to identify genes and pathways in *F. sacchari*. From an evolutionary perspective, the origin of fungal gibberellin biosynthesis is independent of that in plants [29]. The earlier speculation was that fungi acquired GA biosynthetic genes from higher plants via horizontal gene transfer [30]. However, the function of gibberellins in fungi is less well understood, although they can influence the growth and development of their host plants [31]. During the infection of the host plants, gibberellins contribute to the ability of *F. fujikuroi* to grow invasively in the symplasts of the parenchyma cells of the rice epidermis to confer a selective advantage [9]. Thus, the symbiosis of gibberellins produced by endophytic fungi could be a promising strategy to overcome the adverse effects of abiotic stresses on plants [32]. In the current study, several intermediates and final biosynthetic steps are catalyzed by *P450-2* and *DES* (i.e., synthesis of GA9, GA25, GA12, GA13, or GA7), such that the relative accumulation of GA4 and GA7 in NaNO_3_ or (NH_4_)_2_SO_4_ media could be due to the increased expression of these two genes. However, the up-regulation of a given GA gene was not always associated with the high production levels of the corresponding GAs. For example, NaNO_3_ or (NH_4_)_2_SO_4_ media induced the high-level expression of 13-hydroxylase (*P450-3*), which catalyzes the hydroxylation on C-13 of GA4 or GA7 to GA3 in the last steps of the GA biosynthetic pathway [14]. Additionally, the gibberellin gene cluster expression was repressed by growth in urea relative to growth on NaNO_3_ or (NH_4_)_2_SO_4_. This repression could be associated with a dramatic decrease in the metabolic flux of gibberellins, a metabolic bottleneck that occurs downstream of GA4 and GA7 formation and upstream of ent-kaurene formation. This metabolic bottleneck is due, at least in part, to the nitrogen-dependent regulation of GA gene cluster expression. Accumulating evidence indicates that the production of bioactive gibberellins in fungi is tightly regulated by developmental and environmental cues [33]. Nevertheless, the modulation of the expression of the entire gibberellin gene cluster, including the bilateral flanking genes, is complex and requires additional study. Global regulatory proteins and miRNAs are also required to regulate secondary metabolite production [28].

Nitrogen fertilizers are commonly applied in farming to increase crop yield, but they also modify the ability of several plants to resist pathogen infections. Nitrogen availability might play an essential role in plant diseases by enhancing fungal cell growth and influencing the expression of genes required for successful pathogenesis. In this study, these integrated analyses allow us to uncover additional information for a more comprehensive understanding of biological events relevant to fungal secondary metabolic regulation in response to nitrogen availability. These findings can thus provide essential information for *Fusarium* management in the field, although further experiments are necessary to confirm whether such a scenario exists for *F. sacchari* that causes PBD in sugarcane.

## 4. Materials and Methods

### 4.1. Fungal Strain and Culture Conditions

*F. sacchari* CNO-1 (FsCNO-1) was isolated in 2012 from sugarcane plants exhibiting PBD symptoms in Guangxi, China. The isolate was maintained on a slant medium containing potato dextrose agar (PDA) at 4 °C, until use. FsCNO-1 spores (1 mL, 1.0 × 10^6^ conidia/mL) were inoculated into 200 mL Erlenmeyer flasks containing 60 mL Czapek medium (per liter: K_2_HPO_4_ 1 g, MgSO_4_·7H_2_O 0.5 g, KCl 0.5 g, Fe_2_SO_4_ 0.01 g, Sucrose 30 g) supplemented with 3 g/L of NaNO_3_, (NH_4_)_2_SO_4_ or urea as the nitrogen source. The flasks were shaken on a rotary shaker at 200 rpm and 28 °C for three days. Mycelia from the different nitrogen cultures were harvested by centrifugation at 8000 rpm for 15 min at 4 °C for the immediate extraction of total RNA.

### 4.2. Transcriptomic Analysis

Total RNA was extracted from FsCNO-1 grown in the three nitrogen mediums (NaNO_3_, (NH_4_)_2_SO_4_, and urea) using RNAprep Pure plant Kit (Tiangen Biotech, Beijing, China). The quality and quantity of RNA were assessed using Bioanalyzer (Agilent Technologies, Santa Clara, CA, USA). Libraries were produced using an Illumina TruSeq RNA Sample Prep Kit (Illumina, San Diego, CA, USA). The library products were sequenced using the Illumina Hiseq 2500 platform in Majorbio company (Majorbio, Shanghai, China).

RNA-seq reads were trimmed using the SeqPrep (https://github.com/stjohn/SeqPrep) (accessed on 13 June 2019) and Sickle v1.33 software. The remaining clean reads were mapped against 14,679 FsCNO-1 annotated gene models using HISAT2 v2.1.0 [34]. The gene expression level was calculated for quantification based on the fragments per kilobase of exon per million fragments mapped (FPKMs) using Cufflinks [35]. Furthermore, the DEGs were identified using DESeq2 (|log2Fold Change| ≥ 1 and FDR < 0.01) [36]. GO enrichment and KEGG pathway analysis were performed using the ggplot2 [37].

### 4.3. In Silico Analysis of PKS/NRPS-, SM-, and CAZYme-Associated Genes

FsCNO-1 protein sequences were searched against the Natural Product Domain Seeker (NaPDoS) polyketide synthesis/nonribosomal peptide synthetase (PKS/NRPS) database v2.0 (http://napdos.ucsd.edu/napdos_home.html) (accessed on 3 May 2020) [38]. The putative PKS/NRPS protein sequences were further searched against the PKS/NRPS website prediction tool v1.1 (http://nrps.igs.umaryland.edu/) (accessed on 30 April 2020) for the confirmation of specific domains [39]. To identify gene clusters and core genes for the potential secondary metabolite biosynthesis in FsCNO-1, BLAST [40] was performed with the *F. verticillioides* 7600, *F. oxysporum* 4278 [41], and *F. fujikuroi* IMI 58289 genomes [9], respectively. Based on InterProScan v94.0 software, cytochrome P450 was compared using the Interpro database [42]. Carbohydrate-Active EnZymes database (CAZYme) [43] (through the software hmmer v3.3.2) was used for the functional annotation of carbohydrate enzyme genes [44]. Adobe Illustrator was used to drawing micro collinear vector diagrams of functional gene clusters based on their gene sequence and position in the genome.

### 4.4. Quantitative RT-PCR

Total RNA was extracted and reverse-transcribed in a 20 μL reaction volume using the PrimeScript^TM^ RT reagent Kit with gDNA Eraser (Takara, Dalian, China). qRT-PCR analysis was performed three times for each extracted RNA sample using SYBR^®^ Premix Ex Taq™ (Tli RNaseH Plus) (Takara, Dalian, China) according to the manufacturer’s protocol. PCR amplifications were performed using a Roche Light Cycler 480 (Indianapolis, IN, USA). cDNA was synthesized from each sample, and RNA levels were normalized to β-actin levels of FsCNO-1. A relative quantitative method (2^−ΔΔCt^) was used to evaluate the quantitative variation [45]. Primer sequences are listed in Table S4.

### 4.5. Extraction and Analysis of Gibberellins

Gibberellic acids were extracted from 200 mL culture filtrate after culturing for three days using ion exchange resins as previously described [46]. The experiments were carried out using a static method wherein 5 g D301G and D201I exchange resin (NKU, Tianjin, China) were added to 200 mL culture filtrate at pH 2.5 and 3.5, respectively. The exchange resins were shaken for 10 h to achieve saturation and improve gibberellic acid extraction. The gibberellic acid was then eluted with 10 mL acetone and dried in a Vacufuge concentrator (Eppendorf, Hamburg, Germany). The residue was dissolved in HPLC-grade methanol for UHPLC-MS/MS analysis. A 1290 series UHPLC system coupled with a 6460 Triple Quadrupole (QqQ) mass spectrometer (Agilent Technologies, Waldbronn, Germany) was used for gibberellin analysis. An aliquot (1.0 μL) of each solution containing gibberellins was injected into the UHPLC equipped with a Hypersil GOLD C18 column (1.9 µm, 2.1 × 100 mm, Thermo Scientific, Waltham, MA, USA). For MS/MS detection, the ionization source parameters in positive and negative modes were the same as that described in the literature [47]. A linear gradient from 20% to 100% acetonitrile in 10 mM HCOOH over 30 min was used for elution. The flow rate was 0.3 mL/min. The extracted samples and standard mixture of gibberellins (GA3, GA4, and GA7, Sigma-Aldrich, St. Louis, MO, USA) were injected under the same conditions for UHPLC analysis. Each run was repeated three times, and the detector response was measured in the peak area.

## 5. Conclusions

In summary, integrated transcriptome and UHPLC-MS/MS metabolite analysis indicated that nitrogen affected multiple steps in the gibberellin synthesis and metabolic flux to impact the net and relative accumulation of gibberellic acid compounds. Additionally, the transcriptome results illuminated the potential effects of nitrogen availability on the expression of several genes involved in fungal toxin synthesis or that encode PKS/NRPS, cytochrome P450, and carbohydrate-active enzymes, which expanded our knowledge of the role that nitrogen sources play in both secondary metabolite biosynthesis.

## Figures and Tables

**Table 1:** Influence of nitrogen on mycotoxin synthesis expression in FsCNO-1.

Mycotoxin Synthesis	Gene_ID/Description	FPKM			log2FC		
		NaNO_3_	Urea	(NH_4_)_2_SO_4_	Urea vs. NaNO_3_	(NH_4_)_2_SO_4_ vs. NaNO_3_	Urea vs. (NH_4_)_2_SO_4_
Fusarubin	FSAC_04059 polyketide synthase	1.66	3.62	5.57	−1.12	−1.75	0.62
	FSAC_04742 methyltransferase	0.55	1.01	1.51	−0.88	−1.46	0.58
	FSAC_03718 monooxygenase	0.17	0.33	0.02	−0.96	3.09	−4.04
	FSAC_09095 dehydrogenase	0.20	0.15	0.54	0.42	−1.43	1.85
	FSAC_12585 reductase	0.77	1.03	3.66	−0.42	−2.25	1.83
	FSAC_09765 transcription factor	0.12	0.00	0.37	−3.06	−1.62	−1.43
Bikaverin	FSAC_04364 polyketide synthase	0.01	0.01	0.96	0	−6.58	6.58
	FSAC_09746 hypothetical protein	0.37	0.66	8.22	−0.83	−4.47	3.64
	FSAC_00970 o-methyltransferase	0.08	0.19	9.72	−1.25	−6.92	5.68
	FSAC_03389 transcription factor	7.01	2.52	323.19	1.48	−5.53	7.00
	FSAC_09877 transcription factor	1.30	1.02	22.08	0.35	−4.09	4.44
	FSAC_11009 efflux pump	1.56	0.67	124.02	1.22	−6.31	7.53
Fusaric_acid	FSAC_02491 polyketide synthase	921.40	1699.88	496.25	−0.88	0.89	−1.78
	FSAC_08022 aspartokinase	1066.35	1586.00	445.86	−0.57	1.26	−1.83
	FSAC_08313 dihydrofolate reductase	5615.34	10,397.00	2127.52	−0.89	1.40	−2.29
	FSAC_02703 o-acetyltransferase	1077.10	2977.85	406.74	−1.47	1.40	−2.87
Fusarin C	FSAC_14605 polyketide synthase/NRPS	6.87	47.48	9.05	−2.79	−0.40	−2.39
	FSAC_10601 hydrolase	132.87	270.61	260.08	−1.02	−0.97	−0.06
	FSAC_07424 elongation factor 1-gamma	46.03	102.39	199.67	−1.15	−2.12	0.96
	FSAC_09464 hypothetical protein	9.19	39.23	18.16	−2.09	−0.98	−1.11
	FSAC_09939 oxidoreductase	22.35	74.63	111.84	−1.74	−2.32	0.58
	FSAC_10936 hypothetical protein	47.74	134.21	90.51	−1.49	−0.92	−0.57
	FSAC_12527 oxidoreductase	14.15	52.27	40.10	−1.89	−1.50	−0.38
	FSAC_08908 hypothetical protein	86.96	358.76	184.48	−2.04	−1.09	−0.96
	FSAC_10763 methyltransferase	1.85	85.71	0.00	−5.53	0.89	−6.42

**Table 2:** Influence of nitrogen on cytochrome P450 expression in FsCNO-1.

Gene ID	Gene Name	Function Class/Group	FPKM			log2FC		
			NaNO_3_	Urea	(NH_4_)_2_SO_4_	Urea vs. NaNO_3_	(NH_4_)_2_SO_4_ vs. NaNO_3_	Urea vs. (NH_4_)_2_SO_4_
FSAC_07416	TRI13	Secondary metabolism/ trichothecene biosynthesis	1.04	0.03	2.96	5.12	−1.51	6.62
FSAC_07004	TRI13	Secondary metabolism/ trichothecene biosynthesis	181.85	272.37	301.56	−0.58	−0.73	0.15
FSAC_02438	STCS	Secondary metabolism/ sterigmatocystin biosynthesis	4.93	6.64	4.80	−0.43	0.04	−0.47
FSAC_07378	STCS	Secondary metabolism/ sterigmatocystin biosynthesis	0.75	1.99	0.93	−1.41	−0.31	−1.10
FSAC_03140	Bph	Xenobiotic metabolism/ benzoate para hydroxylase	0.04	0.06	0.16	−0.58	−2.00	1.42
FSAC_06106	Bph	Xenobiotic metabolism/ benzoate para hydroxylase	0.93	0.68	13.73	0.45	−3.88	4.34
FSAC_12735	CYP52A3	Xenobiotic metabolism/ alkane inducible P450	0.31	1.01	0.15	−1.70	1.05	−2.75
FSAC_01874	Pdm	Defense against host secreted factors/Pisatin demethylase	1.16	2.73	3.24	−1.23	−1.48	0.25
FSAC_10736	Pdm	Defense against host secreted factors/Pisatin demethylase	9.35	17.04	41.21	−0.87	−2.14	1.27
FSAC_07317	Pdm	Defense against host secreted factors/Pisatin demethylase	2.97	6.25	15.65	−1.07	−2.40	1.32
FSAC_13209	Pdm	Defense against host secreted factors/Pisatin demethylase	1.01	1.29	4.41	−0.35	−2.13	1.77

## Data Availability

All data generated or analyzed during this study were included in this published article and its Supplementary Information files. Further inquiries can be directed to the corresponding author.

## Supplementary Materials

The following supporting information can be downloaded at: https://www.mdpi.com/article/10.3390/ijms241310832/s1.
