# Supplementary material for: Integration of Transcriptomic and Metabolomic Profiles Provides Insights into the Influence of Nitrogen on Secondary Metabolism in Fusarium sacchari
Source: Int J Mol Sci. 2023 Jun 29;24(13):10832. doi: 10.3390/ijms241310832 (PMC10341903; doi:10.3390/ijms241310832)
Supplement: Supplementary file 1 [file ijms-24-10832-s001.zip › ijms-2367580-supplementary.pdf]

Supplementary Figures:

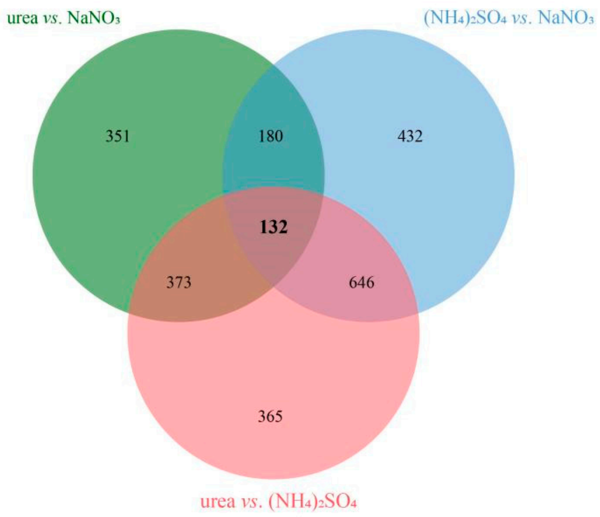

Figure S1. Venn diagram shows the DEGs in different nitrogen sources.

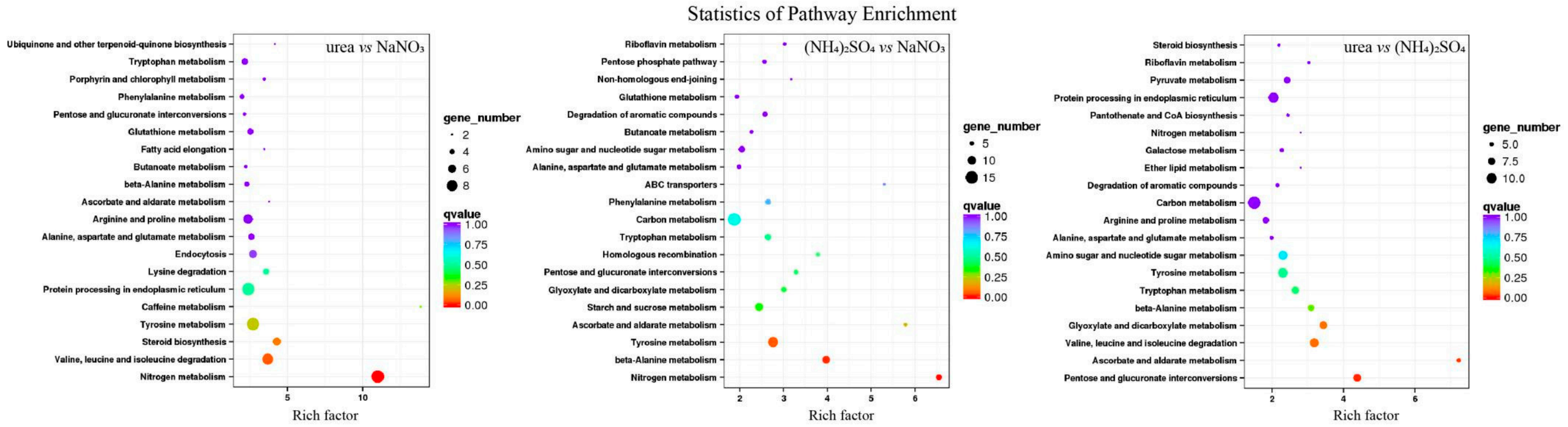

Figure S2. KEGG pathway enrichment analysis of different nitrogen source related- DEGs.

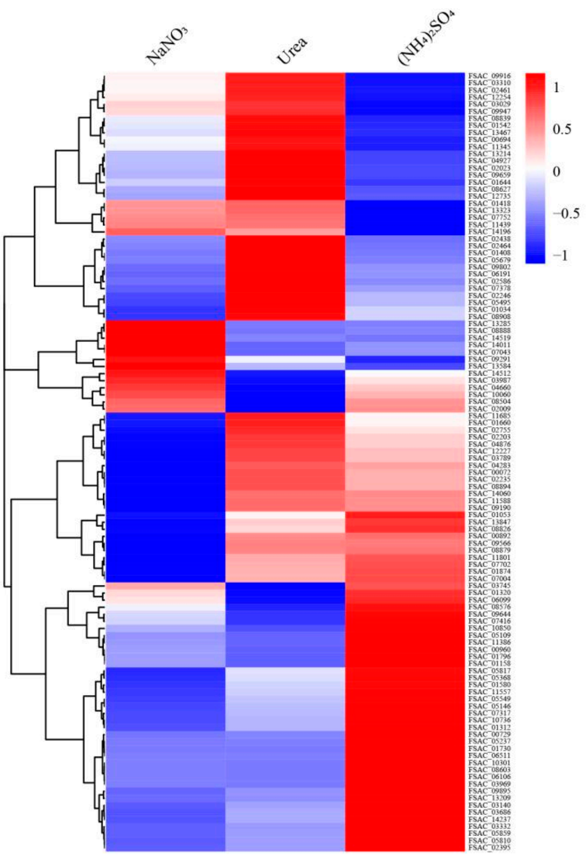

Figure S3. Heatmap clustering analysis of differentially expressed genes related to cytochrome P450.

Supplementary Tables:

Table S1. Summary of top 20 DEGs in each of the KEGG pathways were highly enriched.

	Gene ID	Description	FPKM		Log2FC
			$NaNO_3$	Urea	
	FSAC_03237	NADP-specific glutamate dehydrogenase	100.36	7.37	2.97
	FSAC_03649	Nitrate reductase	4.85	33.73	-2.83
	FSAC_07111	Nitronate monooxygenase	27.67	157.50	-2.58
	FSAC_07254	Glutamine synthetase	110.55	31.61	1.75
	FSAC_07565	Nitrate transporter	82.96	5.98	3.12
	FSAC_10020	Nitrite reductase	426.37	15.46	4.56
	FSAC_12335	Formamidase	2.38	0.43	2.34
	FSAC_13390	Nitrate reductase	155.02	20.29	2.88
**urea *vs.* $NaNO_3$**	FSAC_14519	Cytochrome P450	233.24	13.36	4.08
	FSAC_00621	2-oxoisovalerate dehydrogenase E1	9.50	58.43	-2.67
	FSAC_03650	Aldehyde dehydrogenase (NAD+)	8.07	62.72	-3.01
	FSAC_06079	Related to enoyl-CoA hydratase	32.99	120.65	-1.93
	FSAC_09278	Aldehyde dehydrogenase	87.01	4.80	4.12
	FSAC_10236	2-oxoisovalerate dehydrogenase	8.44	86.85	-3.36
	FSAC_13109	2-oxoisovalerate dehydrogenase E2	5.94	35.92	-2.60
	FSAC_13290	Lambda-crystallin	3.95	0.87	2.01
	FSAC_13775	3-methylcrotonyl-CoA carboxylase	14.43	94.22	-2.76
	FSAC_01067	C-14 sterol reductase ERG-3	9.25	37.66	-1.87
	FSAC_02464	Cytochrome P450	6.98	29.05	-2.10
	FSAC_03102	TGL4-triacylglycerol lipase	29.97	5.53	2.37
	**Gene ID**	**Description**	**FPKM**		**Log2FC**
			$NaNO_3$	$(NH_4)_2SO_4$	
	FSAC_03649	Nitrate reductase	4.85	1.06	2.12
	FSAC_07254	Glutamine synthetase	110.55	26.98	2.01
	FSAC_07565	Nitrate transporter	82.96	7.85	2.90
	FSAC_10020	Nitrite reductase	426.37	13.18	4.85
	FSAC_12335	Formamidase	2.38	0.58	2.02
	FSAC_13390	Nitrate reductase	155.02	14.68	3.39
	FSAC_14519	Cytochrome P450	233.24	4.45	5.71
	FSAC_01450	Copper amine oxidase	3.17	0.61	2.29
**$(NH_4)_2SO_4$ *vs.* $NaNO_3$**	FSAC_01604	UGA1-4-aminobutyrate aminotransferase	11.85	70.19	-2.53
	FSAC_02595	Copper amine oxidase	1.89	9.83	-2.37
	FSAC_03809	Peroxisomal amine oxidase	0.09	1.49	-3.65
	FSAC_04309	Glutamate decarboxylase	147.39	1236.63	-3.02
	FSAC_07363	Corticosteroid-binding protein	31.12	4.62	2.74
	FSAC_08532	Pantoate-beta-alanine ligase	10.17	33.87	-1.74
	FSAC_09278	Aldehyde dehydrogenase	87.01	293.69	-1.75
	FSAC_10912	Copper amine oxidase	0.59	2.32	-1.93
	FSAC_01348	hypothetical protein	6.78	1.27	2.35
	FSAC_01450	Copper amine oxidase	3.17	0.61	2.29
	FSAC_02311	Alcohol dehydrogenase	0.15	1.15	-2.66
	FSAC_02595	Amine oxidase	1.89	9.83	-2.37
	**Gene ID**	**Description**	**FPKM**		**Log2FC**
			$(NH_4)_2SO_4$	urea	
	FSAC_00591	L-arabinitol 4-dehydrogenase	40.74	5.34	2.74
	FSAC_00955	L-galactonate dehydratase	14.11	2.31	2.56
**urea *vs.***	FSAC_02877	UDP-glucose 6-dehydrogenase	27.57	168.32	-2.27
**$(NH_4)_2SO_4$**	FSAC_03319	Endopolygalacturonase	2.02	0.00	4.57
	FSAC_13314	Endo-polygalacturonase	12.21	0.16	5.77
	FSAC_02877	Probable UDP-glucose 6-dehydrogenase	27.57	168.32	-2.27
	FSAC_03650	Aldehyde dehydrogenase	6.73	62.72	-3.26
	FSAC_06313	Aldehyde dehydrogenase	180.55	42.31	2.06

	FSAC_06795	Gluconolactonase	0.10	12.98	-6.65
	FSAC_09278	Aldehyde dehydrogenase	293.69	4.80	5.88
	FSAC_00621	2-oxoisovalerate dehydrogenase E1	9.96	58.43	-2.58
	FSAC_01604	UGA1-4-aminobutyrate aminotransferase	70.19	5.11	3.67
	FSAC_10236	Dehydrogenase E1 component	10.40	86.85	-3.05
	FSAC_13109	2-oxoisovalerate dehydrogenase E2	6.75	35.92	-2.38
	FSAC_01927	Related to ATP-binding protein	15.90	65.67	-2.03
	FSAC_02038	Malate dehydrogenase	821.61	147.43	2.50
	FSAC_03197	Probable formate dehydrogenase	439.08	27.59	3.95
	FSAC_07462	Glycine cleavage system H protein	169.02	717.74	-2.40
	FSAC_08232	Peroxisomal catalase	51.06	0.66	6.15
	FSAC_09716	Catalase	7.57	38.67	-2.40

Table S2. Presence of PKS/NRPS in FsCNO-1 and other reference strains.

Gene	Secondary metabolite	*F. sacchari* CNO-1	*F. fujikuroi* IMI 58289	*F. verticillioides* 7600	*F. oxysporum* 4287	Description
			**Polyketide synthases**			
PKS 1/NRPS	Equisetin/Trichosetin	FSAC_01355	FFUJ_02219	FVEG_12610	FOXG_15296	probable polyketide synthase
PKS 2		FSAC_10615	FFUJ_00118	FVEG_00079	n.p.	polyketide synthase
PKS 3	Fusarubin	FSAC_04059	FFUJ_03984	FVEG_03695	FOXG_05816	polyketide synthase
PKS 4	Bikaverin	FSAC_04364	FFUJ_06742	FVEG_03379	FOXG_04757	polyketide synthase
PKS 5		n.p.	n.p.	n.p.	FOXG_10805	probable polyketide synthase
PKS 6	Fusaric acid	FSAC_02491	FFUJ_02105	FVEG_12523	FOXG_15248	polyketide synthase
PKS 7		FSAC_13741	FFUJ_06260	FVEG_01914	FOXG_03051	polyketide synthase
PKS 8	Fujikurins	FSAC_00846	FFUJ_12090	FVEG_10497	FOXG_11892	Polyketide synthase
PKS 9/NRPS		FSAC_02291	FFUJ_14695	FVEG_11932	FOXG_03945	related to polyketide synthase
PKS 10/NRPS	Fusarin C	FSAC_14605	FFUJ_10058	FVEG_11086	n.p.	polyketide synthase/NRPS
PKS 11	Fumonisin	n.p.	FFUJ_09241	FVEG_00316	n.p.	peptidsynthase condensation domain
PKS 12		FSAC_10998	FFUJ_10347	FVEG_13715	FOXG_16513	probable polyketide synthase
PKS 12a-1		n.p.	n.p.	n.p.	FOXG_14850	probable polyketide synthase
PKS 12a-2		n.p.	n.p.	n.p.	FOXG_15886	probable polyketide synthase
PKS 13	Gibepyrones	FSAC_07220	FFUJ_12020	FVEG_10535	FOXG_11954	polyketide synthase
PKS 14		FSAC_10909	FFUJ_11034	FVEG_08425	FOXG_10070	probable polyketide synthase
PKS 15		FSAC_13430	n.p.	FVEG_05537	n.p.	probable polyketide synthase
PKS 16		n.p.	FFUJ_11199	n.p.	n.p.	probable type I polyketide synthase
PKS 17		FSAC_05628	FFUJ_12066	n.p.	n.p.	probable type I polyketide synthase
PKS 18	Asperfuranone/Chaetoviridin	FSAC_01471	FFUJ_12074	n.p.	n.p.	polyketide synthase
PKS 19	Fujikurins	n.p.	FFUJ_12239	n.p.	n.p.	probable polyketide synthase
PKS 20/NRPS		FSAC_06643	FFUJ_12707	FVEG_13420	FOXG_14587	probable polyketide synthase
PKS 39	Depudecin	n.p.	n.p.	FVEG_01736	FOXG_02884	probable polyketide synthase
PKS 41		n.p.	n.p.	n.p.	FOXG_02741	probable polyketide synthase
		16	18	16	16	
			**Non-ribosomal peptide synthetases**			
NRPS 1	Malonichrom	FSAC_10950	n.p.	FVEG_12503	FOXG_17422	Nonribosomal peptide synthetase
NRPS 2	Ferricrocin (intracell. Sid)	FSAC_06464	FFUJ_04614	FVEG_04296	FOXG_06448	non-ribosomal peptide synthetase
NRPS 3		FSAC_07827	FFUJ_06929	FVEG_03243	FOXG_04898	non-ribosomal peptide synthetase

NRPS 4		FSAC_00673	FFUJ_08113	FVEG_11762	FOXG_13024	related to non-ribosomal peptide synthetase
NRPS 6	Fusarinine (extracell. Sid)	FSAC_03930	FFUJ_10736	FVEG_08697	FOXG_09785	related to AM-toxin synthetase (AMT)
NRPS 10		FSAC_03838	FFUJ_03506	FVEG_05643	FOXG_02458	related to alpha-aminoadipate reductase large subunit
NRPS 11		FSAC_02658	FFUJ_10934	FVEG_08516	FOXG_09998	non-ribosomal peptide synthetase
NRPS 12		FSAC_10897	FFUJ_14790	FVEG_11841	FOXG_13405	non-ribosomal peptide synthetase
NRPS 13		FSAC_11950	FFUJ_02440	FVEG_07777	FOXG_01411	non-ribosomal peptide synthetase
NRPS 17	Ferrichrome	n.p.	FFUJ_03641	FVEG_14029	n.p.	AM-toxin synthetase (AMT)
NRPS 20	Fusaridione A	FSAC_07045	FFUJ_06720	FVEG_03415	FOXG_04709	related to AM-toxin synthetase (AMT)
NRPS 21		FSAC_09574	FFUJ_02022	FVEG_09864	FOXG_10932	non-ribosomal peptide synthetase
NRPS 22	Beauvericin	FSAC_06356	FFUJ_09296	FVEG_09993	FOXG_11847	related to non-ribosomal peptide synthetase
NRPS 23		FSAC_04331	FFUJ_12008	FVEG_10547	FOXG_11967	related to non-ribosomal peptide synthetase
NRPS 24		n.p.	n.p.	FVEG_06502	n.p.	non-ribosomal peptide synthetase
NRPS 25	Acetylaranotin	FSAC_09293	FFUJ_05347	FVEG_13313	n.p.	related to non-ribosomal peptide synthetase
NRPS 26		n.p.	n.p.	FVEG_06496	n.p.	non-ribosomal peptide synthetase
NRPS 31	Apicidin F	n.p.	FFUJ_00003	n.p.	n.p.	non-ribosomal peptide synthetase
NRPS 32	Ferrirhodin-type	n.p.	n.p.	n.p.	FOXG_17272	non-ribosomal peptide synthetase
		15	16	18	15	

Note: n.p. not present in the genome

Table S3. Predicted Cytochrome P450 in FsCNO-1.

Gene ID	Gene Description	DNA Length	Interpro ID	Interpro Description
FSAC_00005	Cytochrome P450 4F4	1691	IPR001128	Cytochrome P450
FSAC_00072	related to cytochrome P450 7B1	2016	IPR001128	Cytochrome P450
FSAC_00226	hypothetical protein TCE0_044f16029	1772	IPR001128	Cytochrome P450
FSAC_00694	hypothetical protein FOTG_07160	1774	IPR001128	Cytochrome P450
FSAC_00719	hypothetical protein FOWG_14704	1707	IPR001128	Cytochrome P450
FSAC_00729	hypothetical protein FOPG_10218	1627	IPR001128	Cytochrome P450
FSAC_00892	related to cytochrome p450	2330	IPR001128	Cytochrome P450
FSAC_00915	related to benzoate 4-monooxygenase cytochrome P450	1675	IPR001128	Cytochrome P450
FSAC_00960	related to benzoate 4-monooxygenase cytochrome P450	2242	IPR001128	Cytochrome P450
FSAC_00966	cytochrome P450 oxidoreductase	1809	IPR001128	Cytochrome P450
FSAC_01034	phenylacetate 2-hydroxylase	2308	IPR001128	Cytochrome P450
FSAC_01053	cytochrome P450 oxidoreductase	9653	IPR001128	Cytochrome P450
FSAC_01158	related to O-methylsterigmatocystin oxidoreductase	1984	IPR001128	Cytochrome P450
FSAC_01312	hypothetical protein FVEG_04308	1978	IPR001128	Cytochrome P450
FSAC_01315	related to Cytochrome P450 3A5	2786	IPR001128	Cytochrome P450
FSAC_01317	hypothetical protein FOQG_05462	1583	IPR001128	Cytochrome P450
FSAC_01320	hypothetical protein FOYG_14910	1640	IPR001128	Cytochrome P450
FSAC_01408	hypothetical protein FOMG_11780	7490	IPR001128	Cytochrome P450
FSAC_01418	hypothetical protein FVEG_12405	3693	IPR001128	Cytochrome P450
FSAC_01422	hypothetical protein FOC4_g10006525	1443	IPR001128	Cytochrome P450
FSAC_01542	Pisatin demethylase	1788	IPR001128	Cytochrome P450
FSAC_01547	related to O-methylsterigmatocystin oxidoreductase	1636	IPR001128	Cytochrome P450
FSAC_01580	related to pisatin demethylase cytochrome P450	1627	IPR001128	Cytochrome P450
FSAC_01644	hypothetical protein FOTG_04497	1897	IPR001128	Cytochrome P450
FSAC_01660	probable cytochrome P450 monooxygenase (lovA)	1862	IPR001128	Cytochrome P450
FSAC_01730	hypothetical protein FVEG_05538	2137	IPR001128	Cytochrome P450
FSAC_01796	hypothetical protein FVEG_13316	3221	IPR001128	Cytochrome P450
FSAC_01874	related to pisatin demethylase cytochrome P450	1681	IPR001128	Cytochrome P450
FSAC_02009	hypothetical protein FOXB_13922	1885	IPR001128	Cytochrome P450
FSAC_02023	related to O-methylsterigmatocystin oxidoreductase	2007	IPR001128	Cytochrome P450
FSAC_02203	hypothetical protein FOVG_17090	1942	IPR001128	Cytochrome P450

FSAC_02235	hypothetical protein FOXB_04367	1878	IPR001128	Cytochrome P450
FSAC_02246	related to TRI13-cytochrome P450	1791	IPR001128	Cytochrome P450
FSAC_02395	hypothetical protein FOC1_g10003456	2145	IPR001128	Cytochrome P450
FSAC_02438	related to sterigmatocystin biosynthesis P450 monooxygenase STCS	3459	IPR001128	Cytochrome P450
FSAC_02461	cytochrome P450 oxidoreductase	1773	IPR001128	Cytochrome P450
FSAC_02464	cytochrome P450, family 51 (sterol 14-demethylase)	1946	IPR001128	Cytochrome P450
FSAC_02586	related to pisatin demethylase cytochrome P450	1723	IPR001128	Cytochrome P450
FSAC_02755	uncharacterized protein FFUJ_07915	3498	IPR001128	Cytochrome P450
FSAC_02775	hypothetical protein FOIG_09507	1893	IPR001128	Cytochrome P450
FSAC_02927	related to benzoate 4-monooxygenase cytochrome P450	2484	IPR001128	Cytochrome P450
FSAC_03029	hypothetical protein FOPG_02369	2472	IPR001128	Cytochrome P450
FSAC_03140	probable benzoate 4-monooxygenase cytochrome P450	1667	IPR001128	Cytochrome P450
FSAC_03310	uncharacterized protein FFUJ_12387	3863	IPR001128	Cytochrome P450
FSAC_03332	hypothetical protein FOMG_15087	1827	IPR001128	Cytochrome P450
FSAC_03686	related to benzoate 4-monooxygenase cytochrome P450	1930	IPR001128	Cytochrome P450
FSAC_03745	hypothetical protein FOWG_12934	1723	IPR001128	Cytochrome P450
FSAC_03789	hypothetical protein FVEG_05542	2388	IPR001128	Cytochrome P450
FSAC_03969	hypothetical protein FVEG_13148	1853	IPR001128	Cytochrome P450
FSAC_03987	gibberellin cluster-C13-oxidase	2411	IPR001128	Cytochrome P450
FSAC_04283	hypothetical protein FOYG_17252	4263	IPR001128	Cytochrome P450
FSAC_04660	related to pisatin demethylase cytochrome P450	2733	IPR001128	Cytochrome P450
FSAC_04678	hypothetical protein FOYG_14294	1867	IPR001128	Cytochrome P450
FSAC_04785	probable cytochrome P450 (involved in C-22 denaturation of the ergosterol side-chain)	2921	IPR001128	Cytochrome P450
FSAC_04876	hypothetical protein FOTG_01974	1757	IPR001128	Cytochrome P450
FSAC_04927	related to cytochrome P450 7A1	1641	IPR001128	Cytochrome P450
FSAC_05109	related to isotrichodermin C-15 hydroxylase (cytochrome P-450 monooxygenase CYP65A1)	2179	IPR001128	Cytochrome P450
FSAC_05146	related to benzoate 4-monooxygenase cytochrome P450	1797	IPR001128	Cytochrome P450
FSAC_05237	related to isotrichodermin C-15 hydroxylase (cytochrome P-450 monooxygenase CYP65A1)	1941	IPR001128	Cytochrome P450
FSAC_05368	related to trichodiene oxygenase cytochrome P450	1699	IPR001128	Cytochrome P450
FSAC_05428	hypothetical protein FOVG_18048	1278	IPR001128	Cytochrome P450
FSAC_05495	related to benzoate 4-monooxygenase cytochrome P450	1631	IPR001128	Cytochrome P450
FSAC_05549	hypothetical protein FVEG_12501	3689	IPR001128	Cytochrome P450
FSAC_05679	related to isotrichodermin C-15 hydroxylase (cytochrome P-450 monooxygenase CYP65A1)	2324	IPR001128	Cytochrome P450
FSAC_05810	hypothetical protein FOXB_09825	1961	IPR001128	Cytochrome P450

FSAC_05817	cytochrome P450 oxidoreductase	2064	IPR001128	Cytochrome P450
FSAC_05859	related to n-alkane-inducible cytochrome P450	1580	IPR001128	Cytochrome P450
FSAC_06099	related to cytochrome P450 monooxygenase (lovA)	1711	IPR001128	Cytochrome P450
FSAC_06106	probable benzoate 4-monooxygenase cytochrome P450	1731	IPR001128	Cytochrome P450
FSAC_06191	hypothetical protein NECHADRAFT_51576	2260	IPR001128	Cytochrome P450
FSAC_06317	hypothetical protein FOZG_14711	1787	IPR001128	Cytochrome P450
FSAC_06406	related to benzoate 4-monooxygenase cytochrome P450	1556	IPR001128	Cytochrome P450
FSAC_06460	hypothetical protein FOMG_04463	1772	IPR001128	Cytochrome P450
FSAC_06511	related to pisatin demethylase cytochrome P450	1976	IPR001128	Cytochrome P450
FSAC_06883	hypothetical protein FOZG_04357	1935	IPR001128	Cytochrome P450
FSAC_06970	hypothetical protein FVEG_06505	1771	IPR001128	Cytochrome P450
FSAC_07004	related to trichodiene oxygenase cytochrome P450	1650	IPR001128	Cytochrome P450
FSAC_07043	bifunctional P-450:NADPH-P450 reductase	4547	IPR001128	Cytochrome P450
FSAC_07317	related to pisatin demethylase	1783	IPR001128	Cytochrome P450
FSAC_07319	related to pisatin demethylase cytochrome P450	1809	IPR001128	Cytochrome P450
FSAC_07373	probable cytochrome P450 51 (eburicol 14 alpha-demethylase)	1737	IPR001128	Cytochrome P450
FSAC_07378	related to sterigmatocystin biosynthesis P450 monooxygenase STCS	2102	IPR001128	Cytochrome P450
FSAC_07416	trichodiene oxygenase	1603	IPR001128	Cytochrome P450
FSAC_07417	related to cytochrome P450 monooxygenase (lovA)	1947	IPR001128	Cytochrome P450
FSAC_07470	related to benzoate 4-monooxygenase cytochrome P450	718	IPR001128	Cytochrome P450
FSAC_07702	related to O-methylsterigmatocystin oxidoreductase	3508	IPR001128	Cytochrome P450
FSAC_07752	hypothetical protein FOCG_07698	1616	IPR001128	Cytochrome P450
FSAC_08105	hypothetical protein FOYG_16165	1802	IPR001128	Cytochrome P450
FSAC_08392	uncharacterized protein FFUJ_01783	2031	IPR001128	Cytochrome P450
FSAC_08504	cytochrome P450 monooxygenase	1920	IPR001128	Cytochrome P450
FSAC_08566	hypothetical protein FOQG_14867	1184	IPR001128	Cytochrome P450
FSAC_08576	hypothetical protein FOVG_04274	2179	IPR001128	Cytochrome P450
FSAC_08603	hypothetical protein OIDMADRAFT_59515	2036	IPR001128	Cytochrome P450
FSAC_08627	hypothetical protein FVEG_05544	1840	IPR001128	Cytochrome P450
FSAC_08637	hypothetical protein FVEG_17280	3251	IPR001128	Cytochrome P450
FSAC_08826	related to cytochrome P450 3A7	1736	IPR001128	Cytochrome P450
FSAC_08839	hypothetical protein FVEG_09294	3650	IPR001128	Cytochrome P450
FSAC_08879	Ent-kaurene oxidase	1906	IPR001128	Cytochrome P450
FSAC_08888	hypothetical protein FOXB_07562	2097	IPR001128	Cytochrome P450

FSAC_08894	hypothetical protein FOYG_14717	2269	IPR001128	Cytochrome P450
FSAC_08908	hypothetical protein FVEG_11079	1644	IPR001128	Cytochrome P450
FSAC_08965	hypothetical protein FVEG_08430	2147	IPR001128	Cytochrome P450
FSAC_09190	hypothetical protein FVEG_13054	1829	IPR001128	Cytochrome P450
FSAC_09291	probable bifunctional P-450:NADPH-P450 reductase	3472	IPR001128	Cytochrome P450
FSAC_09566	related to benzoate-para-hydroxylase (cytochrome P450)	1867	IPR001128	Cytochrome P450
FSAC_09626	related to O-methylsterigmatocystin oxidoreductase	2071	IPR001128	Cytochrome P450
FSAC_09644	GA4 desaturase	3104	IPR001128	Cytochrome P450
FSAC_09659	related to n-alkane-inducible cytochrome P450	1610	IPR001128	Cytochrome P450
FSAC_09802	related to pisatin demethylase	1652	IPR001128	Cytochrome P450
FSAC_09895	hypothetical protein FOYG_16800	2721	IPR001128	Cytochrome P450
FSAC_09916	hypothetical protein FOZG_02590	1626	IPR001128	Cytochrome P450
FSAC_09947	Cytochrome P450 1A1	2539	IPR001128	Cytochrome P450
FSAC_10060	GA14-synthase	1676	IPR001128	Cytochrome P450
FSAC_10301	related to cytochrome P450 monooxigenase	1636	IPR001128	Cytochrome P450
FSAC_10367	Cholesterol 7-alpha-monooxygenase	1711	IPR001128	Cytochrome P450
FSAC_10374	related to TRI13-cytochrome P450	1855	IPR001128	Cytochrome P450
FSAC_10493	hypothetical protein OIDMADRAFT_150154	1417	IPR001128	Cytochrome P450
FSAC_10522	hypothetical protein COCVIDRAFT_39745	2108	IPR001128	Cytochrome P450
FSAC_10689	hypothetical protein FPSE_02370	1893	IPR001128	Cytochrome P450
FSAC_10736	related to pisatin demethylase (cytochrome P450)	1864	IPR001128	Cytochrome P450
FSAC_10850	hypothetical protein FVEG_02379	3275	IPR001128	Cytochrome P450
FSAC_10962	related to trichodiene oxygenase cytochrome P450	1593	IPR001128	Cytochrome P450
FSAC_11008	probable cycloheximide-inducible protein CIP70 (cytochrome P450 family)	1931	IPR001128	Cytochrome P450
FSAC_11192	hypothetical protein FOXB_07498	1713	IPR001128	Cytochrome P450
FSAC_11211	hypothetical protein FOYG_08596	1752	IPR001128	Cytochrome P450
FSAC_11276	hypothetical protein FOVG_17287	1706	IPR001128	Cytochrome P450
FSAC_11345	25-hydroxycholesterol 7-alpha-hydroxylase	1776	IPR001128	Cytochrome P450
FSAC_11386	related to O-methylsterigmatocystin oxidoreductase	2065	IPR001128	Cytochrome P450
FSAC_11439	related to trichodiene oxygenase cytochrome P450	2318	IPR001128	Cytochrome P450
FSAC_11557	hypothetical protein FVEG_04329	1879	IPR001128	Cytochrome P450
FSAC_11588	related to cytochrome P450 7B1	2681	IPR001128	Cytochrome P450
FSAC_11681	unnamed protein product	3013	IPR001128	Cytochrome P450
FSAC_11685	hypothetical protein FOIG_10763	1870	IPR001128	Cytochrome P450

FSAC_11801	cytochrome P450 oxidoreductase	2047	IPR001128	Cytochrome P450
FSAC_11819	related to cytochrome P450 3A7	1969	IPR001128	Cytochrome P450
FSAC_12227	hypothetical protein FOYG_13520	2623	IPR001128	Cytochrome P450
FSAC_12254	related to linoleate diol synthase	3988	IPR001128	Cytochrome P450
FSAC_12624	related to cytochrome P450	2225	IPR001128	Cytochrome P450
FSAC_12669	hypothetical protein FVEG_07101	2618	IPR001128	Cytochrome P450
FSAC_12735	related to n-alkane-inducible cytochrome P450	1730	IPR001128	Cytochrome P450
FSAC_13209	related to pisatin demethylase	1710	IPR001128	Cytochrome P450
FSAC_13214	related to isotrichodermin C-15 hydroxylase (cytochrome P-450 monooxygenase CYP65A1)	1733	IPR001128	Cytochrome P450
FSAC_13285	probable cytochrome P450 51	1573	IPR001128	Cytochrome P450
FSAC_13323	related to cytochrome P450	1768	IPR001128	Cytochrome P450
FSAC_13467	related to cycloheximide-inducible protein CIP70 (cytochrome P450 family)	1562	IPR001128	Cytochrome P450
FSAC_13584	related to isotrichodermin C-15 hydroxylase (cytochrome P-450 monooxygenase CYP65A1)	1754	IPR001128	Cytochrome P450
FSAC_13847	related to pisatin demethylase cytochrome P450	1821	IPR001128	Cytochrome P450
FSAC_13861	probable benzoate 4-monooxygenase cytochrome P450	1588	IPR001128	Cytochrome P450
FSAC_13890	hypothetical protein FOYG_05566	1475	IPR001128	Cytochrome P450
FSAC_13910	Fumitremorgin C monooxygenase-like protein	1265	IPR001128	Cytochrome P450
FSAC_14011	related to pisatin demethylase (cytochrome P450)	1778	IPR001128	Cytochrome P450
FSAC_14060	related to O-methylsterigmatocystin oxidoreductase	1714	IPR001128	Cytochrome P450
FSAC_14196	related to cycloheximide-inducible protein CIP70 (cytochrome P450 family)	1937	IPR001128	Cytochrome P450
FSAC_14237	hypothetical protein FAVG1_12095	1442	IPR001128	Cytochrome P450
FSAC_14512	related to isotrichodermin C-15 hydroxylase (cytochrome P-450 monooxygenase CYP65A1)	1989	IPR001128	Cytochrome P450
FSAC_14519	cytochrome P450 55A1	2512	IPR001128	Cytochrome P450

Table S4. The sequences of the primer sets used for qRT-PCR.

gene ID	Description	Forward	Reverse
FSAC_03987	cytochrome P450 monooxygenase	TCCTGCCAATGTCTGTCTTTC	GGTGGTGATGTGGGTGTATTT
FSAC_08126	copalyl-diphosphate/kaurene synthetase	CCACCGACAAGATCATAGACAG	GCTGAGTGACTCGTGATGATAG
FSAC_05737	geranylgeranyl diphosphate synthase	AGCGACCTTCTCATCAACATC	CAGTCTAAGCTCCCGTTTGTT
FSAC_08504	cytochrome P450 monooxygenase	CATGAAGCCTGGTAGCATAGT	GGGATCATCAAGGCGTCTATT
FSAC_10060	GA14-synthase	CTCATGGACAGCGTCTTGAA	GTGAGTTTGTTCTTGGGTAGGA
FSAC_12979	Ent-kaurene oxidase	GTCTCGCGTCTATTCACACTAC	GCCTTTGACCTCGTCTCTAAG
FSAC_09644	GA4 desaturase	GTGCTACTGACCAAGGCTAATC	GCTATATCACTCCCGTCCTTCT
referencre gene	related actin gene	GAGAACGAGCGTGTCTTGATTGAGCC	TTTCCTCCGCAGAATGAAGAAGGACTC
